# Comparison of three classes of Marginal Risk Set Model in predicting infant mortality among newborn babies at Kigali University Teaching Hospital, Rwanda, 2016

**DOI:** 10.1186/s12887-020-1945-1

**Published:** 2020-02-10

**Authors:** Paul Gatabazi, Sileshi Fanta Melesse, Shaun Ramroop

**Affiliations:** Department of Statistics, University of Kwazulu Natal, Pietermaritzburg, Private Bag X 01, Scottsville, 3209 South Africa

**Keywords:** Infant mortality, Survival analysis, Marginal risk set model, Re-sampling, Covariate, Rwanda

## Abstract

**Background:**

The Infant Mortality Rate (IMR) in Sub-Saharan Africa (SSA) remains the highest relatively to the rest of the world. In the past decade, the policy on reducing infant mortality in SSA was reinforced and both infant mortality and parental death decreased critically for some countries of SSA. The analysis of risk to death or attracting chronic disease may be done for helping medical practitioners and decision makers and for better preventing the infant mortality.

**Methods:**

This study uses popular statistical methods of re-sampling and one selected model of multiple events analysis for measuring the survival outcomes for the infants born in 2016 at Kigali University Teaching Hospital (KUTH) in Rwanda, a country of SSA, amidst maternal and child’s socio-economic and clinical covariates. Dataset comprises the newborns with correct information on the covariates of interest. The Bootstrap Marginal Risk Set Model (BMRSM) and Jackknife Marginal Risk Set Model (JMRSM) for the available maternal and child’s socio-economic and clinical covariates were conducted and then compared to the outcome with Marginal Risk Set Model (MRSM). That was for measuring stability of the MRSM.

**Results:**

The 2117 newborns had the correct information on all the covariates, 82 babies died along the study time, 69 stillborn babies were observed while 1966 were censored. Both BMRSM JMRSM and MRSM displayed the close results for significant covariates. The BMRSM displayed in some instance, relatively higher standard errors for non-significant covariates and this emphasized their insignificance in MRSM. The models revealed that female babies survive better than male babies. The risk is higher for babies whose parents are under 20 years old parents as compared to other parents’ age groups, the risk decreases as the APGAR increases, is lower for underweight babies than babies with normal weight and overweight and is lower for babies with normal circumference of head as compared to those with relatively small head.

**Conclusion:**

The results of JMRSM were closer to MRSM than that of BMRSM. Newborns of mothers aged less than 20 years were at relatively higher risk of dying than those who their mothers were aged 20 years and above. Being abnormal in weight and head increased the risk of infant mortality. Avoidance of teenage pregnancy and provision of clinical care including an adequate dietary intake during pregnancy would reduce the IMR in Kigali.

## Background

The discrepancy in IMR and low life expectancy of the SSA versus the other parts of the world attracts several researchers. The report of the World Bank in 2011 pointed that the IMR was 75/1000 in SSA versus 11/1000 in developed countries [1]. The same report pointed that half of the ten million children who die every year is in SSA. The World Bank dataset from 1960 to 2005 suggests that low life expectancy at birth in SSA is relatively higher in Middle Africa as compared to other sub-regional disparities of SSA [2]. The World Bank records of 2017 indicated that the IMR was 51.50/1000 in SSA [3]. Central African Republic had the highest IMR of 87.60/1000, the lowest IMR were found in Mauritius (11.60/1000), the IMR in Rwanda was 28.90/1000. Several studies on factors that could lower the infant mortality have been done and recommendations were suggested but the IMR remains a problem in SSA.

The multiple events model for infant mortality at the Kigali University Teaching Hospital analysed in [4] leaves a question on whether the adopted model is stable. The main causes of instability may be the correlation of the covariates or relatively small sample size [5]. One of the ways of assessing instability in survival regression models is a use of re-sampling techniques [6]. The analysis in [4] is a none re-sampled model that used the primary dataset of the year 2016. Two observable events per subject are death and the occurrence of at least one of the common conditions that may also cause the long-term death to infants. It was found that the Marginal Risk Set Model (MRSM) also known as the Wei, Lin and Weissfeld Model (WLWM) fit the data well. The WLWM is among the multiplicative methods for analysing ordered events found in [7]. Other multiplicative models include the Andersen-Gill Model (AGM) and the Prentice, Williams and Peterson Model (PWPM) [8].

The present study uses two popular nonparametric methods of re-sampling namely *bootstrap* which is based on the random samples with replacement [9], and *jackknife* method that is based on sampling by leaving out one observation at time [9]. The size of the sample in [4] is 2117 and the record is effective in the year 2016. The long-term results could be assumed according to the stability potentially observed after re-sampling. Several manuscripts on re-sampling in survival analysis are limited on the re-sampled Cox proportional hazards model and on estimating standard errors of the survival and hazard functions such as in [6, 10–13] where bootstrap is involved [13–16]; in which the jackknife is implicated or [17–22] where hazard and survival functions with their respective standard errors are of interest. The present study analyses the bootstrap-based MRSM with 1000 replicates and the jackknife-based MRSM. The results are then compared to that of the MRSM.

## Methods

### Dataset

The time to event data of 2117 newborns at the KUTH is recorded from the 1st January to the 31st December 2016. At KUTH, all newborns are recorded in registries with all details of parents and clinical outcomes of each newborn. The information in registry provides references on card indexes that provide information on clinical behavior of babies after leaving the hospital. KUTH as a site of interest in this study is a central Hospital where most of complicated childbirths countrywide are transferred. In 2016, KUTH recorded relatively high incidence of stillborn cases (69 stillborn babies or 3.259%) and relatively high infant mortality rate (3.873%). Table 1 summarises the information on newborns at KUTH along the study time.

**Table 1:** Summary on newborns under study

Total observations	2117
Deaths during the study time	82 (3.873%)
Stillborn babies	69 (3.259%)
Total events	151 (7.132%)
Censored babies	1966 (92.867%)

The study is interested on subjects with a correct information on the covariates of interests. The two events per subject are observed namely the death and the incidence of at least one chronic disease or complication such as *severe oliguria*, *severe prematurity*, *very low birth weight*, *macrosomia*, *severe respiratory distress*, *gastroparesis*, *hemolytic*, *trisomy*, *asphyxia* and *laparoschisis*. Apart from the *event* status and the *time* to event, 11 covariates are recorded and subdivided in demographic covariates which include the *age* and the place of *residence* for parents; clinical covariates for female parents that include obstetric *antecedents*, type of *childbirth* and previous *abortion*. Clinical covariates for babies include *APGAR*; *gender*, *number* of births at a time, *weight*, circumference of the *head*, and *height*. Table 2 gives a description of the variables of interest.

**Table 2:** Description of variables in the dataset on newborns at Kigali University Teaching Hospital (KUTH) during the period 01-January-2016 to 31-December-2016

Variable	Description	Codes/Values/Unit
Age	Age of parent	0 = under 20, 1 = 20 years old to 34 years old, 2 = 35 years old and above
Residence	Indicator of the residential area of a parent	0 = rural, 1 = urban
Antecedents	Indicator on whether a new born is the first or not	0 = Not the first new born, 1 = first newborn,
Abortion	Indicator on whether a parent aborted previously	0 = not aborted, 1 = aborted once, 2 = aborted more than once
Childbirth Gender	Type of childbirth Gender of a newborn	0 = born using ventouse, 1 = born naturally, 2 = born after surgery 0 = female, 1 = male
Number	Indicator of the number of births at a time	0 = singleton, 1 = multiple
APGAR	Score of *appearance*, *pulse*, *grimaces*, *activity* and *respiration* of a newborn	0 = APGAR less than 4*/*10, 1 = APGAR from 4*/*10 to 6*/*10, 2 = APGAR greater or equal to 7*/*10
Weight	Weight of a newborn	0 = under 2500 *g*, 1 = 2500 *g* to 4500 *g*, 2 = above 4500 *g*
Head	Head circumference of a newborn	0 = below 32 *cm*, 1 = 32 *cm* to 36 *cm*, 2 = above 36 *cm*
Height	Height of a new born	0 = below 46 *cm*, 1 = 46 *cm* to 54 *cm*, 2 = above 54 *cm*
Time	Time from recruitment to study termination	Days
Event	Indicator describing if death occurred during the study time or not	0 = censored, 1 = dead
*n* events	Indicator on the rank of records per subject	1 = first record, 2 = second record

### Statistical methods

#### Marginal risk set model

Assume that *h*(*t*|**x**_*i*_) is the hazard function of the survival time *T* given the *p* fixed covariates **x**_*i*_ = (*x*_*i*1_*, x*_*i*2_*,*. *.*.*, x*_*ip*_). Let *h*_0_(*t*) be the hazard function when **x**_*i*_ = (*0, 0,*. *.*.*, 0*) for all *i*, then

1 $$ h\left(t|{\mathbf{x}}_i\right)={h}_0(t)\ \exp \left({\boldsymbol{\beta}}^{`}{\mathbf{x}}_i\right) $$

where ***β*** = (*β*_1_*, β*_2_*,*. *.*.*, β*_*p*_)^’^ is a *p*-dimensional vector of model parameters [23]. Define an indicator function as.

*δ*_*ij*_(*t*) = 1 if individual *i* is at risk of the *j*^*th*^ event and *δ*_*ij*_(*t*) = 0 otherwise.

The marginal risk set model (MRSM) or the Wei Lin and Weisfeld Model (WLWM) assumes that events are unordered where each event has its own stratum and each data point appears in all strata [4, 24]. In other words, the *k*^th^ time interval per subject is in the *k*^th^ stratum, *k* = 1*,* 2*,*. *.*.*, n*.

The hazard function for the *j*^*th*^ event for the individual *i* is given by

2 $$ h\left(t|{\mathbf{x}}_i\right)={\delta}_{i\ j}(t){h}_{0\ j}(t)\ \exp \left({{\boldsymbol{\beta}}^{`}}_j\ {\mathbf{x}}_i\right) $$

#### Maximum likelihood and parameter estimation

Let]0*, τ*_*i*_ [be the interval of time in which the individual *i* is observed with *n*_*i*_ the number of events of the individual *i* along]0*, τ*_*i*_ [and Assume that two events cannot occur simultaneously in continuous time. The probability density function for the outcome *n*_*i*_ along]0*, τ*_*i*_ [is given by.

*L*(**Φ**) $$ =\prod \limits_{i=1}^n{L}_i\left(\varphi \right) $$

where

3 $$ {L}_i\left(\varphi \right)=\prod \limits_{j=1}^{n_i}h\left(t|{x}_i\right){e}^{-\underset{0}{\overset{\tau_i}{\int }}{\delta}_{ij}(v)h\left(v|{x}_i\right) dv}. $$

In (3), individual *i* has *n*_*i*_ events with *n*_*i*_ ≥ 0 at times *t*_*i*1_ ≤ t_*i*2_ ≤ · · · ≤ t_*ini*_ .

The appropriate partial likelihood functions for tied time to event data is well described in [24] and in [25] and include Breslow’s, Efron’s and Cox’s techniques. The maximum likelihood estimates are given by a system

4 $$ \Big\{{\displaystyle \begin{array}{c}\frac{\partial \ln L\left(\varPhi \right)}{\partial \alpha}\\ {}\frac{\partial \ln L\left(\varPhi \right)}{\partial \beta}\end{array}} $$

where *α* is known as the baseline parameter vector while *β* is a vector of model parameters. The Newton-Raphson method is one of numerical methods used for solving system (4). The adequacy checking of the likelihood estimates is done by finding the elements *ℑ*_*αα*_, *ℑ*_*αβ*_, *ℑ*_*βα*_ and *ℑ*_*ββ*_ of the information matrix *ℑ* and assume that as $n\to \infty, \hat{\varPhi}-\varPhi \mapsto N\left(0,{\Im}^{-1}\left(\hat{\varPhi}\right)\right)$ [4, 26].

In MRSM, *n* is assumed to be the maximum number of events per subject while *τ*_*k*_, *k* = 1*,* 2*, ...n* are times to events per subject along the study time with range [0*, T*]. The study time is partitioned into *n* + 1 intervals of the form

5 $$ 0-{\tau}_1,0-{\tau}_2,...,0-{\tau}_n,0-T. $$

**Fig. 1:**
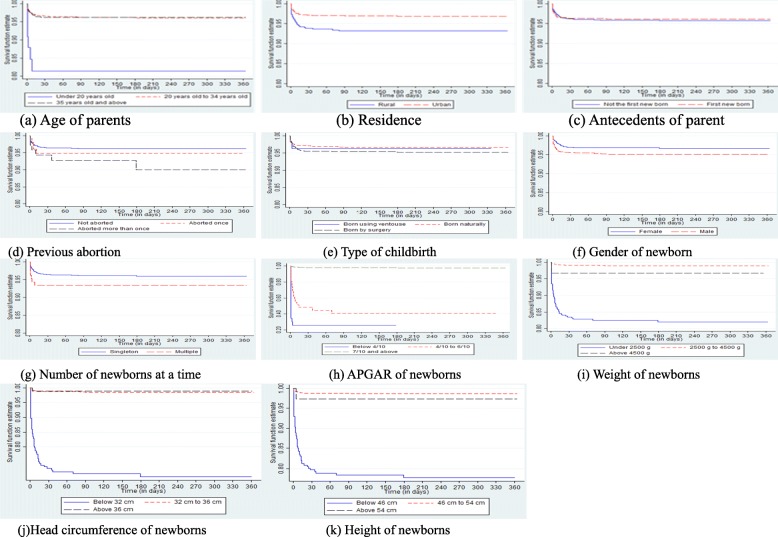
Plots of the survival function per groups of covariates

STATA 15 provides results of the MRSM by applying the Cox Proportional Hazards Model (CPHM) to the dataset in the setup (5). The test of proportional hazards assumption is done by checking patterns of survival functions per groups of each covariate. Figure 1 presents the patterns of survival functions per groups of each covariate using Kaplan-Meier estimation. The patterns are approximately parallel for the covariates of interest. This allows a construction of the MRSM for all the covariates.

### Re-sampled MRSM

The Bootstrap Marginal Risk set Model (BMRSM) is the inference of model (2) based on bootstrap samples (see Appendix). The BMRSM consists of applying model (2) to each of the *B* bootstrap samples **x**_i_^*k^, ∀*k* ∈ [1*, B*] of covariates **x**_*i*_, ∀*i* ∈ [1*, p*]. Bootstrap model parameter estimation in presence of tied events uses either Breslow, Efron or Cox approach. The bootstrap standard error is obtained by using Eq. (6) of the Appendix.

As for the BMRSM, the Jackknife Marginal Risk Model (JMRSM) consists of applying model (2) to each of the *n* jackknife samples **x**_i_^*k^ of covariates **x**_*i*_, *i* ∈ [1*, p*] with a use of Breslow, Efron or Cox approach for estimating the jackknife model parameters. The Jackknife standard error is given by Eq. (7) found in the Appendix.

## Results

Using Breslow estimation [27], Table 3 presents unadjusted MRSM, BMRSM, JMRSM and corresponding adjusted models. Unadjusted and adjusted MRSM, BMRSM and JMRSM are also presented in Tables 4 and 5 for Efron [28] and Cox estimation [29].

**Table 3:** Breslow estimation

MRSM						BMRSM				JMRSM			
Covariate (reference)	Level	HR	SE	*P* > z	95% CI	HR	SE	*P* > z	95% CI	HR	SE	*P* > z	95% CI
Age (Under 20 years old)	20 to 34 years old	0.277	0.100	*p <* 0*.*001	[0.137; 0.560]	0.277	0.088	*p <* 0*.*001	[0.149; 0.515]	0.277	0.081	*p <* 0*.*001	[0.155; 0.493]
	35 years old and above	0.395	0.157	0.020	[0.181; 0.863]	0.395	0.132	0.005	[0.205; 0.761]	0.395	0.127	0.004	[0.210; 0.741]
Residence (Rural)	Urban	0.847	0.139	0.309	[0.614; 1.167]	0.847	0.148	0.341	[0.601; 1.193]	0.847	0.158	0.372	[0.587; 1.220]
Antecedents (Not 1st newborn)	1st newborn	0.806	0.157	0.270	[0.550; 1.182]	0.806	0.138	0.207	[0.577; 1.126]	0.806	0.134	0.193	[0.582; 1.116]
Abortion (Not aborted)	Aborted once	1.405	0.398	0.231	[0.806; 2.448]	1.405	0.459	0.298	[0.741; 2.664]	1.405	0.471	0.311	[0.728; 2.710]
	Aborted more than once	0.479	0.161	0.028	[0.248; 0.925]	0.479	0.280	0.208	[0.152; 1.507]	0.479	0.360	0.328	[0.110; 2.094]
Childbirth (Ventouse)	Natural	0.873	0.491	0.808	[0.290; 2.627]	0.873	1.973	0.952	[0.010; 73.427]	0.873	0.329	0.718	[0.416; 1.829]
	Surgery	1.115	0.613	0.843	[0.380; 3.274]	1.115	2.517	0.962	[0.013; 93.040]	1.115	0.372	0.744	[0.580; 2.143]
Gender (Female)	Male	1.740	0.296	0.001	[1.247; 2.429]	1.740	0.324	0.003	[1.209; 2.505]	1.740	0.337	0.004	[1.191; 2.544]
Number (Singleton)	Multiple	0.409	0.131	0.005	[0.218; 0.766]	0.409	0.107	0.001	[0.245; 0.682]	0.409	0.100	*p <* 0*.*001	[0.252; 0.661]
APGAR (Below 4/10)	4/10 to 6/10	0.377	0.112	0.001	[0.211; 0.673]	0.377	0.127	0.004	[0.195; 0.729]	0.377	0.139	0.008	[0.182; 0.778]
	7/10 and above	0.130	0.036	*p <* 0*.*001	[0.076; 0.222]	0.130	0.033	*p <* 0*.*001	[0.079; 0.212]	0.130	0.031	*p <* 0*.*001	[0.081; 0.208]
Weight (Under 2500 g)	2500 g to 4500 g	0.250	0.068	*p <* 0*.*001	[0.146; 0.427]	0.250	0.064	*p <* 0*.*001	[0.151; 0.412]	0.250	0.063	*p <* 0*.*001	[0.153; 0.408]
	Above 4500 g	0.442	0.285	0.206	[0.125; 1.565]	0.442	4.002	0.928	[0.000; 2.290 × 10^7^]	0.442	0.508	0.478	[0.046; 4.222
Head (Below 32 cm)	32 cm to 36 cm	0.456	0.128	0.005	[0.263; 0.789]	0.456	0.115 0	0.002	[0.277; 0.749]	0.456	0.117	0.002	[0.275; 0.753]
	Above 36 cm	0.290	0.219	0.102	[0.066; 1.278]	0.290	4.156	0.931	[0.000; 4.470 × 10^11^]	0.290	0.284	0.206	[0.043; 1.971]
Height (Below 36 cm)	46 cm to 54 cm	0.894	0.276	0.716	[0.488; 1.637]	0.894	0.241	0.677	[0.527; 1.516]	0.894	0.253	0.692	[0.513; 1.557]
	Above 54 cm	1.670	1.264	0.498	[0.379; 7.361]	1.670	22.884	0.970	[0.000; 7*.*73 × 10^11^]	1.670	1.612	0.596	[0.251; 11.093]
Adjusted MRSM						Adjusted BMRSM				Adjusted JMRSM			
Covariate (reference)	Level	HR	SE	*P* > z	95% CI	HR	SE	*P* > z	95% CI	HR	SE	*P* > z	95% CI
Age (Under 20 years old)	20 to 34 years old	0.307	0.107	0.001	[0.155; 0.609]	0.309	0.089	*p <* 0*.*001	[0.176; 0.543]	0.309	0.083	*p <* 0*.*001	[0.182; 0.523]
	35 years old and above	0.472	0.179	0.047	[0.225; 0.992]	0.489	0.145	0.016	[0.274; 0.874]	0.489	0.137	0.011	[0.282; 0.848]
Abortion (Not aborted)	Aborted once	1.482	0.406	0.152	[0.866; 2.537]	–	–	–	–	–	–	–	–
	Aborted more than once	0.541	0.175	0.057	[0.287; 1.019]	1.607	- 0.304	- 0.012	- [1.109; 2.328]	–	–	–	–
Gender (Female)	Male	1.672	0.280	0.002	[1.204; 2.321]	0.417	0.106	0.001	[0.254; 0.686]	1.607	0.316	0.016	[1.093; 2.363]
Number (Singleton)	Multiple	0.401	0.128	0.004	[0.214; 0.750]	0.412	0.137	0.008	[0.215; 0.791]	0.417	0.103	*p <* 0*.*001	[0.258; 0.677]
APGAR (Below 4/10)	4/10 to 6/10	0.414	0.119	0.002	[0.236; 0.726]	0.150	0.034	*p <* 0*.*001	[0.096; 0.234]	0.412	0.142	0.010	[0.210; 0.809]
	7/10 and above	0.144	0.038	*p <* 0*.*001	[0.086; 0.242]	0.240	0.057	*p <* 0*.*001	[0.151; 0.381]	0.150	0.033	*p <* 0*.*001	[0.098; 0.232]
Weight (Under 2500 g)	2500 g to 4500 g	0.238	0.060	*p <* 0*.*001	[0.144; 0.391	0.478	4.519	0.938	[0.000; 5.32 × 10^7^]	0.240	0.057	*p <* 0*.*001	[0.151; 0.381]
	Above 4500 g	0.447	0.284	0.205	[0.129; 1.550	0.439	0.103	*p <* 0*.*001	[0.277; 0.696]	0.478	0.419	0.400	[0.086; 2.669]
Head (Below 32 cm)	32 cm to 36 cm	0.420	0.100	*p <* 0*.*001	[0.264; 0.669]	0.303	4.200	0.931	[0.000; 1.970 × 10^11^]	0.439	0.107	0.001	[0.273; 0.707]
	Above 36 cm	0.284	0.210	0.089	[0.067; 1.211]					0.303	0.298	0.225	[0.044; 2.084]
		*χ* ^2^ = 213*.*161, *p <* 0*.*001				*χ* ^2^ = 203*.*14, *p <* 0*.*001				*χ* ^2^ = 22*.*310, *p <* 0*.*001

**Table 4:** Efron estimation

MRSM						BMRSM				JMRSM			
Covariate (reference)	Level	HR	SE	*P* > z	95% CI	HR	SE	*P* > z	95% CI	HR	SE	*P* > z	95% CI
Age (Under 20 years old)	20 to 34 years old	0.230	0.083	*p <* 0*.*001	[0.114; 0.466]	0.230	0.086	*p <* 0*.*001	[0.111; 0.478]	0.230	0.083	*p <* 0*.*001	[0.114; 0.466]
	35 years old and above	0.324	0.129	0.005	[0.149; 0.706]	0.324	0.128	0.004	[0.149; 0.703]	0.324	0.125	0.004	[0.152; 0.691]
Residence (Rural)	Urban	0.831	0.137	0.261	[0.602; 1.147]	0.831	0.160	0.337	[0.570; 1.212]	0.831	0.174	0.376	[0.552; 1.252]
Antecedents (Not 1st newborn)	1st newborn	0.756	0.149	0.156	[0.513; 1.113]	0.756	0.149	0.155	[0.514; 1.112]	0.756	0.143	0.140	[0.521; 1.096]
Abortion (Not aborted)	Aborted once	1.393	0.396	0.244	[0.798; 2.430]	1.393	0.470	0.326	[0.719; 2.699]	1.393	0.522	0.377	[0.668; 2.904]
	Aborted more than once	0.452	0.154	0.020	[0.232; 0.880]	0.452	0.322	0.265	[0.112; 1.826]	0.452	0.391	0.359	[0.083; 2.465]
Childbirth (Ventouse)	Natural	0.736	0.408	0.580	[0.249; 2.179]	0.736	1.482	0.879	[0.014; 38.109]	0.736	0.336	0.502	[0.301; 1.801]
	Surgery	0.921	0.499	0.880	[0.319; 2.661]	0.921	1.858	0.968	[0.018; 47.963]	0.921	0.388	0.846	[0.403; 2.104]
Gender (Female)	Male	1.823	0.312	*p <* 0*.*001	[1.304; 2.549]	1.823	0.361	0.002	[1.238; 2.687]	1.823	0.400	0.006	[1.186; 2.804]
Number (Singleton)	Multiple	0.324	0.106	0.001	[0.170; 0.617]	0.324	0.100	*p <* 0*.*001	[0.177; 0.591]	0.324	0.096	*p <* 0*.*001	[0.181; 0.578]
APGAR (Below 4/10)	4/10 to 6/10	0.214	0.065	*p <* 0*.*001	[0.118; 0.387]	0.214	0.080	*p <* 0*.*001	[0.102; 0.447]	0.214	0.093	*p <* 0*.*001	[0.091; 0.501]
	7/10 and above	0.070	0.020	*p <* 0*.*001	[0.041; 0.121]	0.070	0.019	*p <* 0*.*001	[0.041; 0.120	0.070	0.019	*p <* 0*.*001	[0.041; 0.119]
Weight (Under 2500 g)	2500 g to 4500 g	0.231	0.063	*p <* 0*.*001	[0.135; 0.395]	0.231	0.064	*p <* 0*.*001	[0.134; 0.396]	0.231	0.062	*p <* 0*.*001	[0.136; 0.391]
	Above 4500 g	0.412	0.269	0.174	[0.115; 1.479]	0.412	3.892	0.925	[0.000; 4.57 × 10^7^]	0.412	0.485	0.451	[0.041; 4.149]
Head (Below 32 cm)	32 cm to 36 cm	0.422	0.119	0.002	[0.243; 0.734]	0.422	0.115	0.002	[0.247; 0.720]	0.422	0.118	0.002	[0.244; 0.729]
	Above 36 cm	0.246	0.187	0.065	[0.055; 1.093]	0.246	3.784	0.927	[0.000; 3.030 × 10^12^]	0.246	0.251	0.169	[0.033; 1.819]
Height (Below 36 cm)	46 cm to 54 cm	0.917	0.285	0.781	[0.499; 1.687]	0.917	0.290	0.784	[0.494; 1.704]	0.917	0.294	0.788	[0.489; 1.721]
	Above 54 cm	1.692	1.283	0.488	[0.383; 7.476]	1.692	24.567	0.971	[0.000; 3.890 × 10^12^]	1.692	1.700	0.601	[0.236; 12.140]
Adjusted MRSM						Adjusted BMRSM				Adjusted JMRSM			
Covariate (reference)	Level	HR	SE	*P* > z	95% CI	HR	SE	*P* > z	95% CI	HR	SE	*P* > z	95% CI
Age (Under 20 years old)	20 to 34 years old	0.262	0.092	*p <* 0*.*001	[0.132; 0.522]	0.265	0.088	*p <* 0*.*001	[0.138; 0.509]	0.265	0.088	*p <* 0*.*001	[0.138; 0.508]
	35 years old and above	0.407	0.155	0.018	[0.193; 0.859]	0.421	0.151	0.016	[0.208; 0.850]	0.421	0.146	0.013	[0.213; 0.833]
Abortion (Not aborted)	Aborted once	1.487	0.408	0.149	[0.868; 2.546]	–	–	–	–	–	–	–	–
	Aborted more than once	0.520	0.170	0.046	[0.274; 0.987]	- 1.684	- 0.336	- 0.009	- [1.138; 2.490]	–	–	–	–
Gender (Female)	Male	1.764	0.297	0.001	[1.268; 2.453]	0.322	0.097	*p <* 0*.*001	[0.178; 0.583]	1.684	0.367	0.017	[1.098; 2.582]
Number (Singleton)	Multiple	0.308	0.101	*p <* 0*.*001	[0.162; 0.586]	0.246	0.093	*p <* 0*.*001	[0.117; 0.515]	0.322	0.101	*p <* 0*.*001	[0.175; 0.594]
APGAR (Below 4/10)	4/10 to 6/10	0.249	0.073	*p <* 0*.*001	[0.140; 0.442]	0.085	0.021	*p <* 0*.*001	[0.052; 0.138]	0.246	0.100	0.001	[0.110; 0.546]
	7/10 and above	0.081	0.022	*p <* 0*.*001	[0.048; 0.137]	0.225	0.057	*p <* 0*.*001	[0.137; 0.369]	0.085	0.021	*p <* 0*.*001	[0.052; 0.138]
Weight (Under 2500 g)	2500 g to 4500 g	0.222	0.057	*p <* 0*.*001	[0.135; 0.366]	0.487	5.083	0.945	[0.000; 3.730 × 10^8^]	0.225	0.056	*p <* 0*.*001	[0.138; 0.367]
	Above 4500 g	0.430	0.276	0.189	[0.122; 1.512]	0.403	0.105	*p <* 0*.*001	[0.242; 0.671]	0.487	0.453	0.440	[0.078; 3.023]
Head (Below 32 cm)	32 cm to 36 cm	0.388	0.093	*p <* 0*.*001	[0.243; 0.622]	0.252	3.678	0.925	[0.000; 6.680 × 10^11^]	0.403	0.108	0.001	[0.238; 0.683]
	Above 36 cm	0.235	0.175	0.052	[0.054; 1.014]					0.252	0.259	0.180	[0.034; 1.889]
		*χ* ^2^ = 203*.*061, *p <* 0*.*001				*χ* ^2^ = 172*.*14, *p <* 0*.*001				*χ* ^2^ = 21*.*514, *p <* 0*.*001

**Table 5:** Cox estimation

MRSM						BMRSM				JMRSM			
Covariate (reference)	Level	HR	SE	*P* > z	95% CI	HR	SE	*P* > z	95% CI	HR	SE	*P* > z	95% CI
Age (Under 20 years old)	20 to 34 years old	0.193	0.085	*p <* 0*.*001	[0.081; 0.458]	0.193	0.094	0.001	[0.074; 0.502]	0.193	0.088	*p <* 0*.*001	[0.079; 0.472]
	35 years old and above	0.267	0.128 *p <* 0*.*001	0.006	[0.104; 0.682]	0.267	0.131	0.007	[0.102; 0.697]	0.267	0.124	0.004	[0.107; 0.662]
Residence (Rural)	Urban	0.766	0.150	0.175	[0.521; 1.126]	0.766	0.221	0.356	[0.435; 1.349]	0.766	0.221	0.356	[0.435; 1.350]
Antecedents (Not 1st newborn)	1st newborn	0.763	0.185	0.264	[0.475; 1.226]	0.763	0.219	0.345	[0.435; 1.338]	0.763	0.194	0.289	[0.463; 1.258]
Abortion (Not aborted)	Aborted once	1.404	0.453	0.293	[0.746; 2.643]	1.404	0.627	0.448	[0.585; 3.369]	1.404	0.593	0.422	[0.613; 3.215]
	Aborted more than once	0.378	0.152	0.015	[0.172; 0.830]	0.378	0.336	0.274	[0.066; 2.155]	0.378	0.446	0.409	[0.038; 3.814]
Childbirth (Ventouse)	Natural	0.732	0.481	0.635	[0.202; 2.653]	0.732	0.369	0.537	[0.273; 1.968]	0.732	0.365	0.532	[0.276; 1.945]
	Surgery	1.016	0.654	0.980	[0.288; 3.590]	1.016	0.480	0.973	[0.403; 2.565]	1.016	0.455	0.971	[0.423; 2.443]
Gender (Female)	Male	1.991	0.405	0.001	[1.336;2.966]	1.991	0.534	0.010	[1.177; 3.368]	1.991	0.601	0.023	[1.101; 3.599]
Number (Singleton)	Multiple	0.218	0.111	0.003	[0.080; 0.589]	0.218	0.155	0.033	[0.054; 0.882]	0.218	0.131	0.011	[0.067; 0.709]
APGAR (Below 4/10)	4/10 to 6/10	0.080	0.042	*p <* 0*.*001	[0.029; 0.224]	0.080	0.056	*p <* 0*.*001	[0.020; 0.319]	0.080	0.052	*p <* 0*.*001	[0.022; 0.287]
	7/10 and above	0.021	0.011	*p <* 0*.*001	[0.008; 0.056]	0.021	0.014	*p <* 0*.*001	[0.006; 0.076]	0.021	0.011	*p <* 0*.*001	[0.008; 0.061]
Weight (Under 2500 g)	2500 g to 4500 g	0.236	0.070	*p <* 0*.*001	[0.131; 0.423]	0.236	0.077	*p <* 0*.*001	[0.124; 0.448]	0.236	0.068	*p <* 0*.*001	[0.134; 0.415]
	Above 4500 g	0.378	0.257	0.153	[0.100; 1.436]	0.378	4.696	0.938	[0.000; 1.410 × 10^10^]	0.378	0.473	0.437	[0.033; 4.386]
Head (Below 32 cm)	32 cm to 36 cm	0.391	0.119	0.002	[0.216; 0.708]	0.391	0.101	*p <* 0*.*001	[0.236; 0.649]	0.391	0.115	0.001	[0.219; 0.698]
	Above 36 cm	0.212	0.171	0.055	[0.043; 1.033]	0.212	3.376	0.922	[0.000; 7.780 × 10^12^]	0.212	0.238	0.167	[0.023; 1.913]
Height (Below 36 cm)	46 cm to 54 cm	0.828	0.283	0.582	[0.423; 1.620]	0.828	0.254	0.539	[0.454; 1.512]	0.828	0.284	0.582	[0.423; 1.622]
	Above 54 cm	1.706	1.351	0.500	[0.361; 8.060]	1.706	28.569	0.975	[0.000; 3.090 × 10^14^]	1.706	1.747	0.602	[0.229; 12.707]
Adjusted MRSM						Adjusted BMRSM				Adjusted JMRSM			
Covariate (reference)	Level	HR	SE	*P* > z	95% CI	HR	SE	*P* > z	95% CI	HR	SE	*P* > z	95% CI
Age (Under 20 years old)	20 to 34 years old	0.218	0.094	*p <* 0*.*001	[0.094; 0.509]	0.219	0.078	*p <* 0*.*001	[0.109; 0.439]	0.219	0.087	*p <* 0*.*001	[0.101; 0.476]
	35 years old and above	0.341	0.157	0.019	[0.138; 0.841]	0.352	0.133	0.006	[0.167; 0.738]	0.352	0.141	0.009	[0.160; 0.771]
Abortion (Not aborted)	Aborted once	1.479	0.459	0.208	[0.804; 2.719]	–	–	–	–	–	–	–	–
	Aborted more than once	0.424	0.161	0.024	[0.201; 0.892]	- 1.833	- 0.544	- 0.041	- [1.025; 3.278]	–	–	–	–
Gender (Female)	Male	1.886	0.374	0.001	[1.278; 2.783]	0.227	0.136	0.013	[0.070; 0.732]	1.833	0.528	0.036	[1.042; 3.225]
Number (Singleton)	Multiple	0.214	0.108	0.002	[0.079; 0.576]	0.091	0.053	*p <* 0*.*001	[0.029; 0.286]	0.227	0.135	0.013	[0.070; 0.730]
APGAR (Below 4/10)	4/10 to 6/10	0.098	0.050	*p <* 0*.*001	[0.036; 0.267]	0.026	0.013	*p <* 0*.*001	[0.010; 0.067]	0.091	0.062	*p <* 0*.*001	[0.024; 0.345]
	7/10 and above	0.026	0.012	*p <* 0*.*001	[0.010; 0.066]	0.215	0.060	*p <* 0*.*001	[0.125; 0.371]	0.026	0.013	*p <* 0*.*001	[0.010; 0.069]
Weight (Under 2500 g)	2500 g to 4500 g	0.213	0.057	*p <* 0*.*001	[0.125; 0.361]	0.398	4.183	0.930	[0.000; 3.590 × 10^8^]	0.215	0.057	*p <* 0*.*001	[0.128; 0.362]
	Above 4500 g	0.364	0.245	0.134	[0.097; 1.364]	0.374	0.102	*p <* 0*.*001	[0.219; 0.640]	0.398	0.385	0.340	[0.060; 2.650]
Head (Below 32 cm)	32 cm to 36 cm	0.349	0.090	*p <* 0*.*001	[0.211; 0.579]	0.222	3.684	0.928	[0.000; 7.970 × 10^13^]	0.374	0.105	*p <* 0*.*001	[0.216; 0.648]
	Above 36 cm	0.199	0.160	0.044	[0.042; 0.957]					0.222	0.253	0.186	[0.024; 2.067]
		*χ*^2^ = 200*.*400, *p <* 0*.*001				*χ*^2^ = 190*.*114, *p <* 0*.*001				*χ*^2^ = 23*.*710, *p <* 0*.*001			

The results of the unadjusted JMRSM are relatively close to that of the unadjusted MRSM (Table 3). The standard errors in JMRSM and MRSM are close for all covariates. The standard errors in BMRSM and MRSM are also close for covariates except for all levels of covariates *childbirth* where the standard error in BMRSM is about 4 times that of MRSM and the upper levels of covariates *weight*, *head* and *height* where the standard error in BMRSM is about 20 times that of MRSM. Significance difference in levels of covariates is found at the same covariates for both MRSM, BMRSM and JMRSM except at the upper level of the covariate *abortion* where significance is suggested by the MRSM. Following the recommendations of Parzen and Lipsitz [30], the *χ*^2^ test statistics suggest a higher performance of the JCPHM as compared to the CPHM and BCPHM since the *χ*^2^ is relatively everywhere lower for the JCPHM..

## Discussion

The overall results of MRSM, BMRSM and JMRSM by different approaches of ties handling (Tables 3, 4 and 5) are not critically different as expected. The STATA default method (Breslow) is then of interest in the analysis. The JMRSM is adopted for checking stability since the results are closer to that of MRSM than that of BMRSM. The similarity between MRSM and JMRSM suggests that the MRSM may be stable. The global analysis upholds the significance difference of all levels of covariates *age*, *gender*, *number* and *APGAR* and intermediate levels of covariates *weight* and *head*.

The re-sampled adjusted models by Breslow technique of handling tied events suggest that the risk of death or attracting a chronic disease of babies whose parents’ age range from 20 to 34 years old is lower than that of babies whose parents are under 20 years old and that of babies whose parents are 35 years and above. Basinga et al. [31] argue that the unintended pregnancy induces abortion in Rwanda, their study suggests a relatively higher rate of teenage unintended pregnancies as compared to the other age ranges, this contributes on the first hand, to the increase of infant mortality rate. On the second hand, the study by Olausson et al. [32] confirms a relatively higher risk for teenage pregnancies due to biological immaturity. As for the advanced maternal age, Lampinen et al. [33] point that it is associated with relatively poorer outcomes to pregnancies due to the observed higher incidence of chronic medical conditions among older women.

The results show that the risk for male babies is higher than that of female babies. This complies with the usual better survival outcome of the females as reports several manuscripts such as [34] or [35]. Multiple babies survive better than singleton babies; this is however against the results from studies conducted in Sub-Saharan Africa by Monden and Smits [36] and Pongou et al. [37]. This may be due to the small number of multiple newborns recorded at KUTH along the year 2016. The survival outcomes of babies whose APGAR is below 4/10 are poorer than that of babies with higher APGAR score. Babies whose weight range from 2500 g to 4500 g survive better than those whose weight is below 2500 g and those whose weight is above 4500 g while babies whose circumference of head range from 32 cm to 36 cm survive better than those whose circumference of head is below 32 cm. The results of APGAR, weight and circumference of the head comply with the recommendations of the clinical medicine and related manuscripts such as [38] for example.

The study shows that the BMRSM is close to JMRSM and MRSM for all significant covariate but the BMRSM shows relatively higher standard errors for some non-significant covariates. The discrepancy between standard errors after re-sampling for covariates such as *childbirth*, *weight*, *head* and *height* suggests the instability of the MRSM at these specific covariates and this emphasizes their non-significance in the MRSM.

The present analysis is limited on eleven covariates. Unavailable covariates concerning parents that could improve models are, for example, demographic covariates such as the parent’s education level, employment and income; behavioral covariates namely smoking habit, alcohol consumption and dietary and physiotherapeutic variables such as sports activity level. These variables are not recorded in registry at KUTH.

## Conclusion

Marginal Risk Set Model (MRSM) and related re-sampling using Bootstrap (BMRSM) and Jackknife (JMRSM) are described and compared with a use of the dataset on infant mortality. The JMRSM and MRSM displayed relatively close results. The risk is higher for babies whose parents are under 20 years old parents as compared to older parents. Babies born with APGAR greater or equal to 7/10 were found to have a better survival outcome than those born with APGAR less than 4/10 and those whose APGAR range between 4/10 and 6/10. The risk is lower for underweight babies as compared to babies with normal weight and overweight. The survival outcomes for babies with normal circumference of head were found to be better than those with relatively small head. The study suggests that pregnancy of under 20 years old parents should be avoided, also appropriate clinical ways of keeping pregnancy against any cause of infant abnormality could help in lowering infant mortality.

## Data Availability

The dataset used is confidential. Some information on it is available from authors on reasonable request.

## Appendix

### Bootstrap and Jackknife re-sampling methods

#### Bootstrap

Consider the *p* fixed covariates **x**_*i*_ = (*x*_*i*1_*, x*_*i*2_*,*. *.*.*, x*_*in*_) in Eq. (2) where *x*_*i j,i*∈[1*,p*]_ are independent and identically distributed possibly with distribution *F*_*θ*_ where *θ* is the statistical parameter of interest. Consider the distribution function *F*_*Rn*_ of a random variable *R*_*n*_(**x***, F*_*θ*_). A bootstrap method as described in [9], consists of generating samples.

**x**_***i***_^***^ = **x**_***i***_^**1*^, **x**_***i***_^**2*^, …, **x**_***i***_^**B*^,

where **x**_***i***_^**k*^, k ∈ [1*, B*] are random samples of size *n* drawn with replacement from the sample **x**_*i*_.

The variables of **x**_***i***_^**k*^ are independent and identically distributed with distribution ${\hat{F}}_{\theta, n}$, given **x**; ${\hat{F}}_{\theta, n}$ is an estimator of *F*_*θ*_ from **x**_*i*_; *B* is a number of bootstrap samples also known as replications.

The estimated standard error of the bootstrap statistic of interest is given in Efron and Tibshirani [9] as

6 $$ \hat{s}{e}_B=\sqrt{\frac{1}{B-1}\sum \limits_{b=1}^B{\left[{\hat{\theta}}^{\ast }(b)-\frac{1}{B}\sum \limits_{b=1}^B{\hat{\theta}}^{\ast }(b)\right]}^2} $$

where ${\hat{\theta}}^{\ast }(b)$ is an estimate of the statistic of interest from the *b*^th^ bootstrap sample,

*b* = 1*,* 2*,*. *.*. *.B*

#### Jackknife

Consider the *p* fixed covariates **x**_*i*_ = (*x*_*i*1_*, x*_*i*2_*,*. *.*.*, x*_*in*_) in Eq. (2).

Let *θ* be a statistic of interest. The jackknife samples consist of leaving out one observation at a time, that is *n* samples.

**x**_***i***_^***^ = (*x*_*i*1_*, x*_*i*2_*,*. *.*.*, x*_*i k* − 1_*, x*_*i k* + 1_*,*. *.*.*, x*_*in*_) ∀ *k* ∈ [1*, n*] [9].

The jackknife standard error estimate as propose [9], is

7 $$ \hat{s}{e}_{jack}=\sqrt{\frac{n-1}{n}\sum \limits_{i=1}^n{\left[{\hat{\theta}}^{\ast }(i)-\frac{1}{n}\sum \limits_{i=1}^n{\hat{\theta}}^{\ast }(i)\right]}^2} $$

where *θ*^∗^(*i*), *i* ∈ [1*, n*] is a statistic of interest for the *i*^*th*^ jackknife sample.

## Abbreviations

AGM: Andersen-Gill Model
APGAR: Appearance, Pulse, Grimace, Activity and Respiration
BCPHM: Bootstrap Cox Proportional Hazards Model
BMRSM: Bootstrap Marginal Risk Set Model
CPHM: Cox Proportional Hazards Model
CPHM: Jackknife Cox Proportional Hazards Model
IMR: Infant Mortality Rate
JMRSM: Jackknife Marginal Risk Set Model
KUTH: Kigali University Teaching Hospital
MRSM: Marginal Risk Set Model
PWPM: Prentice, Williams and Peterson Model
SSA: Sub-Saharan Africa
WLWM: Wei, Lin and Weissfeld Model
